# Book Availability Announcement

**DOI:** 10.1120/jacmp.v7i2.2274

**Published:** 2006-05-25

Encyclopedia of Medical Devices and Instrumentation, Second Edition

John G. Webster, Editor

John Wiley and Sons Inc. Publishers, $1,750 USD, 4800 Pages, ISBN 0‐471‐26358‐3, hard cover

John Wiley & Sons has announced the availability of the Encyclopedia of Medical Devices and Instrumentation, Second Edition. The senior editor is John G. Webster and one of the associate editors is Edward S. (Ned) Sternick; a medical physicist with numerous years of experience in the field. The unusual approach of announcing the availability of the six volume reference rather than reviewing it is it's cost; $1,750. The six volumes encompass some 4800 pages covering 260 topics which are authored by over 425 contributors. The major focus is on Biomedical Engineering however some 37 topics deal with Medical Radiation Physics.

Those who wish to examine the table of contents and an illustrative chapter can view them at http://www3.interscience.wiley.com/cgi‐bin/mrwhome/112102158/home

The section I reviewed was titled Biocompatibility of Materials. Regrettably for the Medical Physicists, none of the 37 sections dealing with Medical Radiation Physics were available to review on the web.

James B. Smathers

Book Review Editor JACMP

